# Sero and Carriage Epidemiology of Pertussis in Urban and Rural Regions in Vietnam

**DOI:** 10.3390/vaccines12030225

**Published:** 2024-02-23

**Authors:** Michiko Toizumi, Lien Thuy Le, Hien Anh Thi Nguyen, Thao Thi Thu Le, Noriko Kitamura, Liem Xuan Bui, Nen Minh Ho, Hung Thai Do, Kazunari Kamachi, Nao Otsuka, Minh Xuan Bui, Duc Anh Dang, Lay-Myint Yoshida

**Affiliations:** 1 Department of Pediatric Infectious Diseases, Institute of Tropical Medicine, Nagasaki University, Nagasaki 852-8523, Japan; lmyoshi@nagasaki-u.ac.jp; 2 Department of Microbiology and Immunology, The Pasteur Institute in Nha Trang, Nha Trang 650000, Vietnam; lethuylien250786@gmail.com (L.T.L.); lethuthao121285@gmail.com (T.T.T.L.); hungdt02@yahoo.com (H.T.D.); 3 National Institute of Hygiene and Epidemiology, Hanoi 100000, Vietnam; hienanh75@yahoo.com (H.A.T.N.); dangducanh.nihe@gmail.com (D.A.D.); 4 Center for Surveillance, Immunization, and Epidemiologic Research, National Institute of Infectious Diseases, Tokyo 162-8640, Japan; nkitamur@niid.go.jp; 5 Quang Ngai Provincial Center for Disease Control, Quang Ngai 570000, Vietnam; tcmr.quangngai@gmail.com (L.X.B.); hominhnen2008@gmail.com (N.M.H.); 6 Department of Bacteriology II, National Institute of Infectious Diseases, Tokyo 208-0011, Japan; kamachi@niid.go.jp (K.K.); notsuka@niid.go.jp (N.O.); 7 Khanh Hoa Health Service, Nha Trang 650000, Vietnam; bsminhnt@gmail.com

**Keywords:** pertussis, whooping cough, seroepidemiology, anti-pertussis toxin IgG, vaccine, Vietnam

## Abstract

The underestimation of the pertussis burden prompted our study to investigate the prevalence of recent pertussis infection, its associated factors, and antibody titer changes in the same individuals in Vietnam. Two cross-sectional surveys were conducted in Nha Trang in 2017 and Quang Ngai in 2019, representing high- and low-vaccine-coverage areas, respectively. Serum anti-pertussis toxin immunoglobulin-G (anti-PT IgG) ≥ 62.5 IU/mL by ELISA indicated infection in the previous 12 months. In Nha Trang, the participants of the 2017 survey were followed up in 2019. Logistic regression was used to determine the odds ratios for the characteristics associated with anti-PT IgG ≥ 62.5. The age-stratified prevalence in patients aged >2 years ranged from 2.1% (age 26–35) to 9.6% (3–5) in Nha Trang (2017) and from 7.2% (age 26–35) to 11.4% (6–15) in Quang Ngai. The prevalence tended to be higher in Quang Ngai across all age groups. Cough, recent antibiotic use, and smoking in Nha Trang were positively associated with an anti-PT IgG of ≥62.5, and having been diagnosed with pertussis and persistent cough with paroxysms/whoop in Quang Ngai were positively associated with an anti-PT IgG of ≥62.5. No nasopharyngeal swabs were positive for *Bordetella pertussis* using real-time PCR. The geometric mean of the IgG titer ratio from 2019 to 2017 was 1.45 in the paired samples. This study emphasizes *Bordetella pertussis* circulation across all age groups in both low- and high-vaccine-coverage settings in Vietnam, underscoring the need for continuous and standardized surveillance for a comprehensive understanding of its epidemiology.

## 1. Introduction

Pertussis (whooping cough) is caused by *Bordetella pertussis*, which is an important cause of infant death worldwide. About 24 million pertussis cases and >160,000 deaths in children <5 years of age were estimated in 2014, with the African region and the Southeast Asian region contributing 33% and 26% of cases and 58% and 26% of deaths, respectively [1].

The global incidence of pertussis has declined since vaccine introduction; however, the disease underwent a resurgence, including in some countries that had persistently high vaccination coverage [2]. A shift in cases in older individuals has been reported as well [3]. The global diversity in vaccine types, schedules, coverage, and surveillance systems substantially complicates our understanding of the mechanisms behind the changes in pertussis epidemiology [4].

Among the 10 ASEAN countries, Malaysia and Singapore introduced the diphtheria–pertussis–tetanus vaccine (DPT) in the 1950s, followed by the other countries later in the 1970s and the 1980s. Brunei, Malaysia, and Singapore have switched from whole-cell to acellular vaccines, while the others have yet to do so. All except Cambodia, Laos, Myanmar, and the Philippines have included a booster dose(s) at different times after the primary series of vaccinations. A review summarized the estimated incidence rates based on national passive surveillance, which varied by country, with diverse vaccine coverage. For example, the rate ranged from 0 to 0.0839 per 100,000 people between 2000 and 2017 in the Philippines, where the coverage of one dose and three doses of the DPT vaccine (DPT1 and DTP3) was usually <90%, which is highly likely to be underestimated. The rate ranged from 0 to 38 per 100,000, with a dramatic decrease in 2013 and a consistent DTP1 and DTP3 coverage of over 90% since 2008 in Cambodia. The review concluded that the true burden remains unclear and is likely to be largely underestimated in Southeast Asian countries [5].

In Vietnam, the DPT vaccine was introduced in 1981 and replaced by the pentavalent vaccine (DTP—*Haemophilus influenzae* type B—Hepatitis B) in 2011. The current program uses a whole-cell pertussis vaccine for all doses and includes a primary series of three doses at 2, 3, and 4 months and a booster dose at 18 months. No further boosters are administered after 18 months [6]. The DPT3 coverage has been >90% since 2010, except in 2013 (59%), 2018 (75%), 2019 (89%), and 2021 (83%) [7]. The incidence of pertussis was 84.4 per 100,000 in 1984 and decreased to 0.06–0.11 per 100,000, with 95–108 cases reported, of which more than 50% were infants, in 2012–2013 [6,8]. In 2014, 92 of 102 reported pertussis cases were infants aged less than 6 months [8]. The estimated national incidence increased in 2015 to 0.33 per 100,000 from 0.06–0.12 per 100,000 in 2010–2014 [5]. In 2015, 2016, 2017, 2018, and 2019, 309, 267, 555, 700, and 1013 cases were reported in Vietnam to the WHO repository, respectively, approximately 40% of which were infants aged less than two months who had not received a series of pertussis vaccines [9,10]. Cases are identified and reported based on a clinical or laboratory diagnosis but are likely to be clinically diagnosed at the community level, as laboratory confirmation is not widely available [8]. Such case-reported surveillance is important for assessing disease burden; however, cases are dominated by young children, and the pattern is strongly influenced by age-specific severity and the risk of hospitalization [4]. To track and compare patterns of pertussis infection in a population, regardless of the clinical symptoms or severity, population-based serosurveillance has been used to estimate the incidence of pertussis infection based on the population prevalence of high titers of immunoglobulin G (IgG) antibodies against pertussis toxin (PT) [11], as anti-PT IgG responses occur in most cases of *B. pertussis* infection, and high titers persist only temporarily. Such serosurveillance has not been performed in many low- and middle-income countries, including Vietnam.

This study aimed to estimate the prevalence of recent pertussis infection serologically in a subset of not recently vaccinated children and adults by age groups and its associated factors in a community in Nha Trang, a city, and in Quang Ngai, a remote area in Vietnam. The Quang Ngai survey was additionally conducted, as this area experienced three diphtheria cases in 2019 [12]. Our previous study on diphtheria seroprevalence targeted the same samples as this study, which estimated that the DPT coverage was lower in Quang Ngai than in Nha Trang, as the diphtheria seroprevalence among persons aged 1–5 years was lower in Quang Ngai than in Nha Trang, in addition to the incidence of diphtheria cases. Therefore, we regarded Nha Trang as a high-vaccine-coverage area and Quang Ngai as a low-coverage area [12]. We also investigated waning and acquiring antibodies for the pertussis toxin over a two-year follow-up period and the factors associated with this change. These study findings would be useful for discussing better preventive measures, such as the optimal age for booster vaccinations in Vietnam.

## 2. Materials and Methods

### 2.1. Study Design

A community-based cross-sectional serosurvey of pertussis was conducted in 2017 in Nha Trang. We followed up the pertussis serology of the subjects of the 2017 survey in 2019. In 2019, we conducted another community-based cross-sectional serosurvey in Quang Ngai Province following the occurrence of diphtheria cases in the area.

### 2.2. Study Area and Participants’ Enrollment

Nha Trang is a tourist city in south-central Vietnam with a 2018 population of 426,958 [13]. For the 2017 survey, we targeted two communes in Nha Trang City, Vinh Hai, and Vinh Phuoc, with a total population of 50,061 in 2018. A commune is the smallest Vietnamese administrative unit providing health and educational services. A total of 500 age-stratified individuals were randomly selected from the population data of the communes, 50 from each age stratum (0–5, 6–15, 16–25, 26–35, and 36–55 years) in each commune. All participants of the 2017 survey were invited to participate in a follow-up survey in 2019. The cross-sectional and longitudinal surveys were originally designed as dengue serosurveys, and the methods have been reported in detail elsewhere [14]. We used the samples and information of 510 enrollments in 2017, and 310 of those were followed up in 2019. We especially targeted those aged three years or older among the participants in the surveys to estimate the prevalence of recent pertussis infection serologically to avoid the direct influence of vaccination on the titer.

Another community-based survey was conducted in two districts, Tay Tra and Son Ha, with a population of 99,121 in 2019 in Quang Ngai Province, central Vietnam [15]. It is an area with limited health access due to its mountainous topography, where three diphtheria cases were reported between January and September 2019. A total of 1500 participants were approached for enrollment as follows: 350, 400, 400, and 350 for age groups 1–5, 6–17, 18–40, and 41–55 years, respectively, based on the previously measured diphtheria seroprevalence in Vietnam using multi-stage cluster sampling [12]. This survey was originally conducted for diphtheria serosurveillance. The survey finally recruited 1216 persons, and we used their information and serum samples for this study.

The required sample size for each age group was estimated to be 77 with an 8% precision, assuming the prevalence of recent pertussis infection to be 15% at most [16]; although, the surveys were originally planned for the seroprevalence of other pathogens and we used all available samples from the surveys.

### 2.3. Data Collection and Laboratory Testing

For the community surveys in Nha Trang and Quang Ngai, trained local healthcare workers interviewed the subjects or guardians of the subjects by using a standardized data collection form to collect demographic, clinical, and social information, including sex, age, vaccination history (orally reported), presence of respiratory symptoms in the preceding two weeks, clinical history, travel history, and household information. We also obtained written vaccination records from each participant’s vaccination card or vaccination registration book maintained at the commune health centers in the 2019 surveys. A nasopharyngeal swab sample was collected in the 2017 Nha Trang survey and the 2019 Quang Ngai survey using a nylon-flocked swab that was placed in one milliliter of skim milk–tryptone–glucose–glycerol transport medium. The samples were transported to the Pasteur Institute in Nha Trang and stored at −80 °C until they were tested for *Bordetella pertussis*, *Bordetella parapertussis*, *Bordetella holmesii*, and *Mycoplasma pneumoniae* using multitarget real-time PCR for the insertion sequence IS481 (detection of *B. pertussis* and *B. holmesii*), recA (*B. holmesii*), IS1001 (*B. parapertussis*), and atpD (*M. pneumoniae*) [17].

Serum samples from the surveys in Nha Trang and dried blood samples (DBS) on the Whatman 903 protein saver card (Cytiva, Marlborough, USA) from the survey in Quang Ngai were stored at −80 °C at the Pasteur Institute in Nha Trang. DBS was punched out with a 6 mm hole punch, eluted with 500 μL elution buffer (phosphate-buffered saline), and incubated overnight at 4 °C [12]. The supernatant of the eluted solution of DBS and thawed serum samples were used to measure the level of anti-PT IgG using a commercially available enzyme-linked immunosorbent assay (ELISA) kit (Abcam, Cambridge, UK) following the manufacturer’s protocol. Anti-PT IgG ≥ 62.5 and ≥125 IU/mL were regarded as cut-offs for infection in the preceding 12 months and infection in the preceding 6 months, respectively, as these cut-off levels are often employed in population-based seroepidemiological studies [11,16,18,19,20,21]. Expecting a recent infection, serologically, was considered valid among those who were three years old or older, as they can be regarded as not vaccinated for pertussis for more than one year, which is sufficient to ignore the effect of vaccination on the anti-PT IgG titer [18].

### 2.4. Ethical Considerations

The Institutional Review Boards of the Pasteur Institute in Nha Trang (1775/IPN-DT), Ministry of Health Vietnam (1046/K2DT-KHCN), Institute of Tropical Medicine Nagasaki University (approval number 191226228), and London School of Hygiene and Tropical Medicine (ethics reference number 17518) approved the Quang Ngai survey section of this study. The Institutional Review Board of the Ministry of Health Vietnam (IRB-VN01057-27/2015) approved the Nha Trang survey section of this study. Written informed consent was obtained from all participants or their guardians before enrollment.

### 2.5. Statistical Analysis

The age-stratified prevalence of anti-PT IgG ≥5, ≥62.5, and ≥125 IU/mL and the geometric mean concentration (GMC) were summarized with 95% confidence intervals (CIs) for those who were three years or older using the 2017 Nha Trang and 2019 Quang Ngai surveys. We used the age category used in the 2017 Nha Trang survey for the 2019 Quang Ngai samples for a convenient comparison of the age-stratified prevalence, although we did not statistically compare those between the surveys because the original sampling age category was different in Quang Ngai. We investigated the demographic, clinical, and socioeconomic factors associated with the prevalence of anti-PT IgG ≥ 62.5 IU/mL using logistic regression for those aged three years or more. It was an exploratory analysis for listing the possible risk factors, so we did not adjust for multiplicity. The odds ratio for anti-PT IgG ≥ 62.5 IU/mL for each demographic, clinical, and socioeconomic factor was adjusted for the age group and sex. We described the carriage of *B. pertussis*, *B. parapertussis*, *B. holmesii*, and *M. pneumoniae* in the 2017 Nha Trang and 2019 Quang Ngai surveys.

We evaluated the changes in IgG titers from 2017 to 2019 using the difference in natural log-transformed IgG values, log(IgG2019)–log(IgG2017), in individuals with available paired samples in the Nha Trang surveys. The back-transformation of the log-transformed changes yields the ratios of the geometric means. We investigated the association between each of the demographic, clinical, and socioeconomic factors and log-transformed changes using linear regression. The coefficients were adjusted for the age, sex, and IgG level in 2017 (baseline IgG ≥ 62.5 IU/mL). Additional analysis for the IgG titer change over time was conducted using a mixed-effect linear regression model incorporating random intercept and considering the between-individual variability. We also investigated the association between each characteristic and the (untransformed) ratio of the titers using linear regression. Statistical significance was set at *p* < 0.05. Statistical analyses were conducted using Stata version 17 [22].

## 3. Results

### 3.1. Prevalence of Recent Pertussis Infection in Nha Trang and Quang Ngai

A total of 510 participants (27, 73, 107, 105, 94, and 104 in the age strata of 0–2, 3–5, 6–15, 16–25, 26–35, and 36–55 years, respectively) were enrolled and tested in 2017 in Nha Trang; 220 (43%) were male and 290 (57%) were female. Twenty-seven were aged 0–2 years with a detectable anti-PT IgG of ≥5 IU/mL (81.5%, 95% CI, 62.4–92.1) and a GMC of 27.6 IU/mL (95% CI, 13.8–54.9). Among the participants aged three years or older (*n* = 483), 3–5, 6–15, and 16–25 years of age had a higher prevalence of an anti-PT IgG ≥62.5 IU/mL as follows: 9.6% (95% CI, 4.6–18.8), 5.6% (95% CI, 2.5–11.9), and 6.7% (95% CI, 3.2–13.3), respectively, compared with the 26–35 and 36–55 year age groups, with a prevalence of 2.1% (95% CI, 0.5–8.1) and 3.8% (95% CI, 1.4–9.8), respectively (Table 1). None of the participants were real-time PCR-positive for *B. pertussis*, *B. parapertussis*, or *B. holmesii*. A 13-year-old boy and a 3-year-old girl tested positive for *M. pneumoniae*. The proportions of the anti-PT IgG titers ≥ 62.5 IU/mL were 11.4%, 12.2%, 9.1%, 5.8%, and 6.2% in 3–5, 6–15, 16–25, 26–35, and 36–57 years old, respectively, in Nha Trang, 2019 (age in 2019); however, we did not regard them as the estimated prevalence of recent infection in 2019 because the subjects were part of the 2017 participants and were not sampled from the population appropriately.

A total of 1216 participants (87, 182, 299, 158, 292, and 198 in the 0–2, 3–5, 6–15, 16–25, 26–35, and 36–55 years age groups, respectively) were enrolled and tested in 2019 in Quang Ngai. They were 615 (51%) males and 601 (49%) females. A total of 375 children aged ≤10 years had written vaccination records. Seventy-seven percent of those aged ≤10 years were confirmed to have received at least one dose, and 75% had received three or four doses of DTP. Three (0.3%) and ten (0.9%) reported having ever been diagnosed with pertussis and had a persistent cough, respectively. The proportion of detectable anti-PT antibody ≥5 IU/mL was 87.4% (95% CI, 78.6–92.9), and the GMC was 24.9 IU/mL (95% CI, 19.0–32.7) in 1–2 year-olds. Among the participants aged three years or older (*n* = 1129), all age groups had a relatively similar prevalence of an anti-PT IgG of ≥62.5 IU/mL, ranging from 7.2% to 11.4% (Table 1). None of the participants were real-time PCR-positive for *B. pertussis*, *B. parapertussis*, *B. holmesii*, or *M. pneumoniae*.

### 3.2. Factors Associated with Prevalence of anti-PT IgG ≥ 62.5 IU/mL in Nha Trang and Quang Ngai Surveys

The demographic, clinical, and social characteristics of the participants aged >2 years in the Nha Trang survey in 2017 and the Quang Ngai survey in 2019 are shown in Table 2. Adjusted odds ratios (aORs) for an anti-PT IgG of ≥62.5 IU/mL for each demographic, clinical, and socioeconomic factor among those aged three or older using logistic regression are shown in Table 3 (crude ORs are shown in Supplementary Table S1). In the Nha Trang survey, a cough (aOR, 3.70; 95% CI, 1.52–9.03) and having taken antibiotics (aOR, 3.70; 95% CI, 1.24–11.03) in the preceding two weeks were positively associated with an anti-PT IgG of ≥62.5 IU/mL after adjusting for the age group and sex; each model showed a good fit (*p* = 0.50 and *p* = 0.62, respectively, according to Pearson’s goodness-of-fit test). In the Quang Ngai survey, having been diagnosed with pertussis (aOR, 17.43; 95% CI, 1.54–197.70), a persistent cough with paroxysms/whoop (aOR, 4.27; 95% CI, 1.07–17.00), and smoking (aOR, 2.21; 95% CI, 1.07–4.57) were associated with an anti-PT IgG of ≥62.5 IU/mL. Each model showed a good fit (*p* = 0.18, *p* = 0.32, and *p* = 0.93, respectively, according to Pearson’s goodness-of-fit test). DPT vaccine history, travel history, nursery or school attendance, family with >4 members, and house size were not associated with an anti-PT IgG of ≥62.5 IU/mL in either survey (Table 3).

### 3.3. Change in Anti-PT IgG Titers in Paired Samples from 2017 to 2019

**Figure 1:**
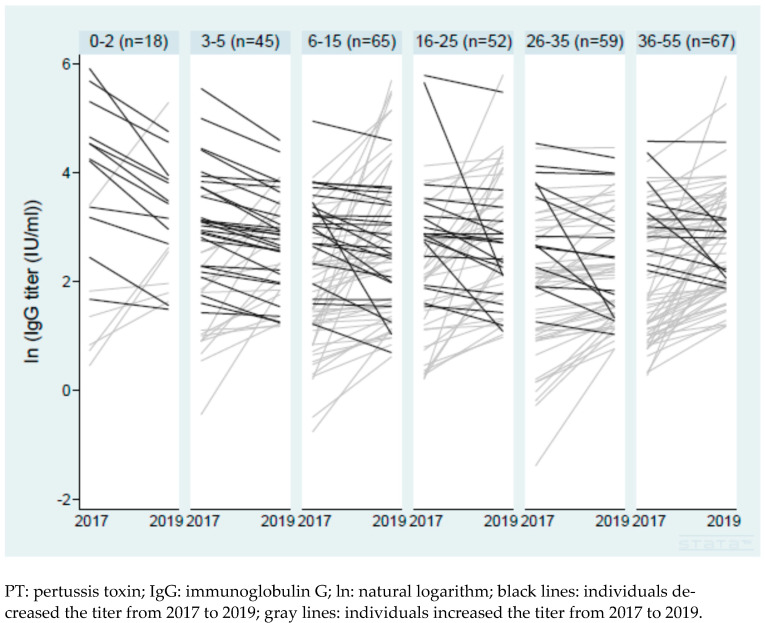
Change in titers of anti-PT IgG from 2017 to 2019 in Nha Trang.

Among the 310 participants we followed up with in Nha Trang in 2019, 306 with paired serum samples taken in 2017 and 2019 were included in the analysis. None of the participants in the 2019 follow-up survey received a pertussis-containing vaccine between the surveys. One hundred and ten participants, aged 0–32 years, were confirmed using written vaccination records. Ninety-four percent of the participants aged 0–5 years had written vaccination records, and 86% of the participants aged 0–5 years had received a confirmed history of three or four doses. Sixty-two percent of the participants aged 6–15 years were confirmed with written records, and 58% of the participants aged 6–15 years were confirmed to be vaccinated with three or four doses. Overall, the IgG titer increased in 199 (65.0%) individuals and with more than a two-fold increase in 79 (25.8%), while it increased only in 33.3% of the 0–2 year-old and 37.8% of the 3–5 year-old groups (Figure 1). The geometric mean of the ratio of the IgG titer in 2019 to the IgG titer in 2017 was 1.45 (95% CI, 1.29–1.62) in all participants, while it was 0.86 (0.48–1.52) in the 0–2 year-olds and 1.11 (0.87–1.42) in the 3–5 year-olds (Table 4). The ratio of IgG titers in the 3–5-year-old group, in those with a higher baseline IgG (≥62.5 IU/mL), and in those with at least one dose of DPT confirmed were smaller compared with the oldest group (age 36–55), those with a lower baseline IgG (<62.5 IU/mL), and those with no DPT history confirmed, respectively (adjusted coefficient −0.43, 95% CI −0.80 to −0.06; adjusted coefficient −1.21, 95% CI −1.69 to −0.73; and adjusted coefficient −0.97, 95% CI −1.83 to −0.12, respectively). In addition, those with a cough in the preceding two weeks in the 2017 survey were associated with a larger ratio of IgG titers in the two years (adjusted coefficient 0.44, 95% CI 0.12–0.76) (Table 4). The crude coefficients are shown in Supplementary Table S2. We used the untransformed ratio of the titers as an outcome and obtained similar results with a high arithmetic mean of the ratio (3.37, 95% CI 2.22–4.51), strong evidence of a positive association with a recent cough in the 2017 survey (adjusted coefficient 6.25, 95% CI 3.01–9.49), and a negative association with confirmed DPT history (−24.34, 95% CI −35.44 to −13.23) (Supplementary Table S3).

A mixed-effect linear regression model considering individual differences also showed a positive log-transformed IgG titer change per year with a coefficient of 0.19 (95% CI 0.13–0.25) (coefficient 2.48, 95% CI −0.45–5.42, for the untransformed IgG titer).

## 4. Discussion

We estimated the prevalence of recent pertussis infections in a population in a city and remote setting in Vietnam. The estimated prevalence of pertussis infection in the prior 12 months in Nha Trang City, Vietnam, 2017, was 9.6% in the 3–5-, 5.6% in the 6–15-, 6.7% in the 16–25-, and 2–4% in the 26–55-year-old groups. The prevalence in adolescents was comparable to that of multinational serosurveillance in Asia (India, Thailand, China, Sri Lanka, Korea, Taiwan, and Japan, 2013–2016), in which 4.8% of individuals aged 10–18 years had an anti-PT IgG of ≥62.5 IU/mL. The prevalence of an anti-PT IgG of ≥62.5 IU/mL among 10–18-year-olds was 6.1% in India, 5.1% in Sri Lanka, 4.4% in Taiwan, and 5.3% in Thailand [16]. Another study in Japan detected approximately 4% of 12–17-year-old students with anti-PT IgG titers of ≥100 EU/mL between 2013 and 2014 [23]. Our study suggests persistent circulation of *B. pertussis* among older children, adolescents, and young adults in Vietnam, as in other Asian countries.

The estimated prevalence of recent pertussis infection in the previous 12 months (anti-PT IgG ≥62.5 IU/mL) was higher in Quang Ngai in 2019 than in Nha Trang in 2017. First, this might be due to the differences in time. Considering the increased IgG titer in the follow-up survey in Nha Trang in 2019, an epidemic may have occurred between 2017 and 2019 in Vietnam. In fact, a higher number of cases was reported to the WHO repository in 2018 (*n* = 700) and 2019 (*n* = 1013) than in 2015 (*n* = 309), 2016 (*n* = 267), and 2017 (*n* = 555) [9], and the incidence was also estimated to be higher in 2017 (0.58/100,000) than in 2016 (0.28/100,000) and 2015 (0.33/100,000) [5]. The proportion of anti-PT IgGs of ≥62.5 IU/mL in Nha Trang 2019 was close to but still lower than that in Quang Ngai in 2019, possibly implying a higher rate of infection in Quang Ngai. However, they were not directly comparable, as the Nha Trang 2019 was not sampled from the population in 2019, but a part of the 2017 participants followed up in 2019. Second, this may be due to differences in place. This study observed a lower proportion of participants with a confirmed history of DPT vaccination in Quang Ngai than that in Nha Trang. In addition, our previous study detected a lower seroprevalence of anti-diphtheria toxoid IgG among children aged 1–5 years in Quang Ngai (36%) than in Nha Trang (68%) [12]. A similar pattern was observed with a lower prevalence of an anti-PT IgG of ≥62.5 IU/mL (29%) among the ages 0–2 years in Quang Ngai compared with that in Nha Trang (48%) in this study. Both could indicate a lower DPT coverage in Quang Ngai, which might subsequently lead to a higher prevalence of pertussis infection. Third, differences in the type of samples, serum, and DBS might have affected the ELISA results. However, this is less likely because we confirmed a good agreement between the ELISA results for anti-diphtheria toxoid IgG titers using serum and DBS [24].

Previous studies have discussed population birth rates, family size, contact patterns, and school attendance as factors of pertussis [25,26], mainly as those of increasing opportunities for transmission. In addition, healthcare provision and socioeconomic factors could be associated with a failure to be vaccinated and, consequently, risk of pertussis [27,28]. In the surveys in Nha Trang and Quang Ngai, families with five or more members, small house size, nursery or school attendance, and confirmed DPT vaccine history were not associated with an IgG of ≥62.5 IU/mL. Cough, taking antibiotics in the preceding two weeks in the Nha Trang survey, having been diagnosed with pertussis, and having had a persistent cough in the Quang Ngai survey were positively associated with an IgG of ≥62.5 IU/mL, which could indicate the reliability of the serological parameter to identify recent pertussis infection. Smoking was also associated with an anti-PT IgG of ≥62.5 IU/mL in the Quang Ngai survey. Smoking has often been discussed as a factor that worsens pertussis symptoms [29,30], while some studies have discussed the possibility of smoking or environmental tobacco smoke increasing the carriage of bacteria in the respiratory tract [31,32]. Smoking or passive smoking might increase not only the severity but also the risk of *B. pertussis* infection.

We could not detect *B. pertussis* in the nasopharyngeal swab samples in the Nha Trang or Quang Ngai surveys. PCR has optimal sensitivity for diagnosing a symptomatic patient with pertussis during the first three weeks of cough but not after five days of antibiotic use [33]. Meanwhile, PCR-positive cases have been reported in asymptomatic epidemiological contacts during outbreak investigations [34,35,36,37]. PCR may not be sufficiently sensitive to detect the later stages of symptomatic or asymptomatic infections in the community between epidemics.

Comparing the anti-PT IgGs in 2017 and 2019 in the same individuals, we observed an overall increase in the titer, which indicated the circulation of *B. pertussis* in the population and an epidemic between the surveys, as discussed above. Cough in the preceding two weeks of the 2017 survey was associated with an increase in IgG titers in these two years. Meanwhile, the titer decreased in those with high baseline IgG titers, suggesting that vaccine-derived or naturally boosted immunity against pertussis toxin is not maintained for long, which is consistent with the observations in the anti-PT IgG titer after pertussis infection, based on which serological estimation of pertussis incidence was established [11]. Those who confirmed receiving DPT were less likely to increase the titer than those not confirmed with a history of DPT, which might suggest a protective effect of the vaccine.

Strengths and limitations: To our knowledge, this is the first study to estimate the prevalence of recent pertussis infections using anti-PT IgG serology in two different settings in Vietnam. We conducted the surveys in a city and in a remote area. However, the two surveys were conducted two years apart, and the sampling methods, including the proportion of age groups, were different; therefore, we could not directly compare the prevalence. It was not possible to estimate the prevalence of recent infection among children aged two years or less, as the serology cannot distinguish between vaccine-derived and naturally boosted immunities. We checked the written vaccination records as much as possible; however, it was difficult to confirm vaccination history, especially in older participants and those who had never been vaccinated. Therefore, the effect of the vaccine on the prevalence of pertussis infection might not be accurately estimated. Considering the vaccine effect using the national DPT3 coverage in Vietnam, the coverage was 88–99% in people born between 1992 and 2019, aged 0–25 in the 2017 Nha Trang and 2019 Quang Ngai surveys, except for a couple of birth cohorts born in 2002 (75%), 2013 (59%), and 2018 (75%) [7]. Most people aged 36–55 years had no DPT vaccination because most of them were born before the DPT introduction, and the 26–35-year-olds had diverse coverage from 0 to 90% due to the transition period. Therefore, the prevalence of recent infection would be higher in those aged 26 years or older if the vaccine could be effective for a long time; however, we did not observe such a difference by age group. Based on the waning anti-PT antibodies after infection [11], an anti-PT IgG of ≥ 62.5 to ≥ 80 IU/mL is often used as the cut-off threshold indicating pertussis infection within 12 months, and cut-off values of ≥ 100 IU/mL and ≥125 IU/mL are used as evidence of a recent infection and acute infection, respectively [38,39]. We used the cut-offs ≥62.5 IU/mL and ≥125 IU/mL as a pertussis infection within 12 months and 6 months, respectively, following previous studies [16,18,19,20,21] so that we can compare our results with those studies. We noted that some studies have used different cut-off values [23,40]; therefore, the prevalence of recent infections would differ by using different cut-off values. However, we believe that our finding of substantial circulation of *B. pertussis* in the community and its risk factors would not change. Finally, we did not impose a multiple-comparison correction to avoid potentially suppressing the identification of genuine associations or trends in the exploratory analysis. Thus, the results are preliminary and require validation through independent studies or confirmatory analyses.

We observed the circulation of *B. pertussis* in a broad age range of the population, especially in older children and young adults, which may indicate the need for an additional booster vaccine in preschool-aged or school-aged children that can suppress circulation to protect infants, who are the most vulnerable, in addition to strengthening the current four doses of DPT. The findings of this study can be applied to other countries or regions with similar backgrounds. However, the epidemiology can change over time and by place, following pertussis epidemics and differences in vaccine coverage, even within a country, as shown by the difference between Nha Trang and Quang Ngai in this study. Therefore, continuous nationwide surveillance using a standardized method is necessary to track the pattern and compare it with other countries and to discuss better preventive measures in Vietnam and similar settings.

## 5. Conclusions

*Bordetella pertussis* circulated across all age groups in both the city and remote areas of Vietnam, even with a high coverage of DPT3. Continuous and standardized surveillance is necessary to understand its epidemiology for better preventive measures in Vietnam and similar settings.

## Figures and Tables

**Table 1:** The proportion of participants with anti-PT IgG ≥ 62.5 and ≥125 IU/mL in each age group in Nha Trang in 2017, and Quang Ngai in 2019.

	Nha Trang in 2017						Quang Ngai in 2019					
Age	N	≥62.5 IU/ml	Prevalence of ≥62.5 IU/Ml (95% CI)	≥125 IU/mL	Prevalence of ≥125 IU/mL (95% CI)	GMC (IU/mL) (95% CI)	N	≥62.5 IU/mL	Prevalence of ≥62.5 IU/mL (95% CI)	≥125 IU/mL	Prevalence of ≥125 IU/mL (95% CI)	GMC (IU/mL) (95% CI)
0−2	27	13	48.1 (30.3−66.4)	4	14.8 (5.7−33.5)	27.6 (13.8−54.9)	87	25	28.7 (20.2−39.1)	10	11.5 (6.3−20.1)	24.9 (19.0−32.7)
3−5	73	7	9.6 (4.6−18.8)	4	5.5 (2.1−13.7)	15.3 (11.6−20.2)	182	18	9.9 (6.3−15.2)	8	4.4 (2.2−8.5)	13.3 (11.4−15.6)
6−15	107	6	5.6 (2.5−11.9)	2	1.9 (0.5−7.2)	9.3 (7.4−11.7)	299	34	11.4 (8.2−15.5)	11	3.7 (2.0−6.5)	15.7 (14.0−17.7)
16−25	105	7	6.7 (3.2−13.3)	3	2.9 (0.9−8.5)	10.4 (8.2−13.3)	158	12	7.6 (4.4−12.9)	3	1.9 (0.6−5.7)	14.5 (12.5−16.9)
26−35	94	2	2.1 (0.5−8.1)	0	NA	8.5 (6.8−10.8)	292	21	7.2 (4.7−10.8)	6	2.1 (0.9−4.5)	15.2 (13.7−16.8)
36−55	104	4	3.8 (1.4−9.8)	0	NA	9.6 (7.9−11.9)	198	19	9.6 (6.2−14.6)	6	3.0 (1.4−6.6)	15.9 (14.1−18.1)

PT: pertussis toxin; IgG: immunoglobulin G; GMC: geometric mean concentration.

**Table 2:** Demographic, clinical, and social characteristics of participants aged >2 years in the Nha Trang survey in 2017 and the Quang Ngai survey in 2019.

Characteristics	Nha Trang in 2017 Number (%)	Quang Ngai in 2019 Number (%)
	*n* = 483	*n* = 1129
**Demographics**		
Age group		
3–5	73 (15.1)	182 (16.1)
6–15	107 (22.2)	299 (26.5)
16–25	105 (21.7)	158 (14.0)
26–35	94 (19.5)	292 (25.9)
36–55	104 (21.5)	198 (17.5)
Sex		
Male	207 (42.9)	558 (49.4)
Female	276 (57.1)	571 (50.6)
**Clinical information: Recent symptoms and medication**		
Respiratory symptom	in proceeding two weeks	in proceeding one month, *n* = 1128
Yes		270 (23.9)
No		858 (76.1)
Cough		
Yes	60 (12.4)	
No	423 (87.6)	
Runny nose		
Yes	58 (12)	
No	425 (88)	
Difficulty breathing		
Yes	2 (0.4)	
No	481 (99.6)	
Took antibiotics		
Yes	28 (5.8)	211 (18.9)
No	455 (94.2)	907 (81.1)
**Clinical information: History and underlying condition**		
Ever diagnosed with Pertussis	*n* = 291 (asked in 2019)	*n* = 1057
Yes	1 (0.3)	3 (0.3)
No	290 (99.7)	1054 (99.7)
Ever had a persistent cough	*n* = 291 (asked in 2019)	*n* = 1073
Yes	8 (2.8)	10 (0.9)
No	283 (97.3)	1063 (99.1)
DPT history orally reported		
At least one dose	204 (42.2)	
No history or unknown	279 (57.8)	
DPT vaccine history confirmed	*n* = 95 (confirmed in 2019)	*n* = 291
At least one dose	90 (94.7)	275 (94.5)
No DPT history	5 (5.3)	16 (5.5)
Going to nursery or school		
Yes	214 (44.3)	382 (33.8)
No	269 (55.7)	748 (66.2)
Chronic disease		
Yes	18 (3.7)	159 (13.7)
No	465 (96.3)	970 (86.3)
Smoking		
Yes	28 (5.8)	155 (13.7)
No	455 (94.2)	974 (86.3)
**Travel history**		
Traveled to another province in Vietnam since 2012	*n* = 291 (asked in 2019)	*n* = 1128
Yes	39 (13.4)	105 (9.3)
No	252 (86.6)	1023 (90.7)
Traveled to another country since 2012	*n* = 291 (asked in 2019)	*n* = 1128
Yes	6 (2.1)	7 (0.6)
No	285 (97.9)	1121 (99.4)
**Household**		
Family with >4 members		
≥5	262 (54.2)	569 (50.4)
1–4	221 (45.8)	561 (49.7)
Family has child(ren) aged <12 years		
Yes	258 (53.4)	995 (88.1)
No	225 (46.6)	135 (12)
Family has smoker(s)		*n* = 1127
Yes	253 (52.4)	659 (58.5)
No	230 (47.6)	468 (41.5)
Family has a member with long-lasting cough (>3 months)		
Yes	3 (0.6)	
No	480 (99.4)	
House size (m^2^)	*n* = 482	*n* = 490
Less than 80	278 (60.0)	438 (89.4)
81 or more	204 (40.0)	52 (10.6)

**Table 3:** Demographic, clinical, and social characteristics of all the participants and those with anti-PT IgG ≥ 62.5 IU/mL, and adjusted odds ratios for anti-PT IgG ≥ 62.5 IU/mL in each characteristic by logistic regression.

	Nha Trang in 2017			Quang Ngai in 2019		
Characteristics	All Number (%)	IgG ≥ 62.5 IU/mL N (%)	Adjusted Odds Ratio *	All Number (%)	IgG ≥ 62.5 IU/mL N (%)	Adjusted Odds Ratio *
	*n* = 483	*n* = 26		*n* = 1129	*n* = 104	
**Demographics**						
Age group						
3–5	73 (15.1)	7 (9.6)	2.43 (0.68–8.70) **	182 (16.1)	18 (9.9)	1.06 (0.54–2.10) **
6–15	107 (22.2)	6 (5.6)	1.32 (0.36–4.89) **	299 (26.5)	34 (11.4)	1.23 (0.68-2.23) **
16–25	105 (21.7)	7 (6.7)	1.70 (0.48–6.02) **	158 (14.0)	12 (7.6)	0.74 (0.35–1.57) **
26–35	94 (19.5)	2 (2.1)	0.55 (0.10–3.07) **	292 (25.9)	21 (7.2)	0.72 (0.38–1.38) **
36–55	104 (21.5)	4 (3.9)	Reference	198 (17.5)	19 (9.6)	Reference
Sex						
Male	207 (42.9)	15 (7.3)	Reference	558 (49.4)	43 (7.7)	Reference
Female	276 (57.1)	11 (4.0)	0.59 (0.26–1.33) ***	571 (50.6)	61 (10.7)	1.49 (0.99–2.25) ***
**Clinical information: Recent symptoms and medication**						
Respiratory symptom	in proceeding two weeks			in proceeding one month, *n* = 1128		
Yes				270 (23.9)	28 (10.4)	0.84 (0.53–1.33)
No				858 (76.1)	76 (8.9)	Reference
Cough						
Yes	60 (12.4)	9 (15.0)	3.70 (1.52–9.03)			
No	423 (87.6)	17 (4.0)	Reference			
Runny nose						
Yes	58 (12)	4 (6.9)	1.18 (0.38–3.61)			
No	425 (88)	22 (5.2)	Reference			
Difficulty breathing						
Yes	2 (0.4)	0 (0)	NA			
No	481 (99.6)	26 (5.4)				
Took antibiotics				in proceeding one month, *n* = 1118		
Yes	28 (5.8)	5 (17.9)	3.70 (1.24-11.03)	211 (18.9)	22 (10.4)	0.84 (0.50–1.38)
No	455 (94.2)	21 (4.6)	Reference	907 (81.1)	80 (8.8)	Reference
**Clinical information: History and underlying condition**						
Ever diagnosed with pertussis	*n* = 291 (asked in 2019)			*n* = 1057		
Yes	1 (0.3)	0 (0.0)	NA	3 (0.3)	2 (66.7)	17.43 (1.54–197.70)
No	290 (99.7)	12 (4.1)		1054 (99.7)	95 (9.0)	Reference
Ever had a persistent cough	*n* = 291 (asked in 2019)			*n* = 1073		
Yes	8 (2.8)	0 (0.0)	NA	10 (0.9)	3 (30.0)	4.27 (1.07–17.00)
No	283 (97.3)	12 (4.2)		1063 (99.1)	98 (9.2)	Reference
DPT history orally reported						
At least one dose	204 (42.2)	12 (5.9)	0.37 (0.10–1.39)			
No history/unknown	279 (57.8)	14 (5.0)	Reference			
DPT vaccine history confirmed	*n* = 95 (confirmed in 2019)			*n* = 291		
At least one dose	90 (94.7)	5 (5.3)	NA	275 (94.5)	30 (10.9)	0.90 (0.19–4.19)
No DPT history	5 (5.3)	0 (0.0)		16 (5.5)	2 (12.5)	Reference
Going to nursery or school						
Yes	214 (44.3)	18 (8.4)	4.2 (0.82–21.49)	382 (33.8)	42 (11.0)	1.07 (0.51–2.24)
No	269 (55.7)	8 (3.0)	Reference	748 (66.2)	62 (8.3)	Reference
Chronic disease						
Yes	18 (3.7)	1 (5.6)	0.81 (0.10–6.51)	159 (13.7)	15 (9.4)	1.09 (0.61–1.97)
No	465 (96.3)	25 (5.4)	Reference	970 (86.3)	89 (9.2)	Reference
Smoking						
Yes	28 (5.8)	2 (7.1)	1.71 (0.31–9.39)	155 (13.7)	16 (10.3)	2.21 (1.07–4.57)
No	455 (94.2)	24 (5.3)	Reference	974 (86.3)	88 (9.0)	Reference
**Travel history**						
Traveled to another province in Vietnam since 2012	*n* = 291 (asked in 2019)			*n* = 1128		
Yes	39 (13.4)	2 (5.1)	0.86 (0.18–4.21)	105 (9.3)	6 (5.7)	1.29 (0.53–3.16)
No	252 (86.6)	10 (4.0)	Reference	1023 (90.7)	98 (9.6)	Reference
Traveled to another country since 2012	*n* = 291 (asked in 2019)			*n* = 1128		
Yes	6 (2.1)	1 (16.7)	0.24 (0.02–2.42)	7 (0.6)	0 (0)	NA
No	285 (97.9)	11 (3.9)	Reference	1121 (99.4)	104 (9.3)	
**Household**						
Family with >4 members						
≥5	262 (54.2)	14 (5.3)	0.98 (0.44–2.23)	569 (50.4)	55 (9.7)	1.09 (0.73–1.64)
1–4	221 (45.8)	12 (5.4)	Reference	561 (49.7)	49 (8.7)	Reference
Family has child(ren) aged <12 years						
Yes	258 (53.4)	13 (5.0)	1.01 (0.44–2.34)	995 (88.1)	94 (9.5)	1.28 (0.64–2.56)
No	225 (46.6)	13 (5.8)	Reference	135 (12)	10 (7.4)	Reference
Family has smoker(s)				*n* = 1127		
Yes	253 (52.4)	15 (5.9)	1.22 (0.54–2.76)	659 (58.5)	63 (9.6)	0.91 (0.60–1.39)
No	230 (47.6)	11 (4.8)	Reference	468 (41.5)	41 (8.8)	Reference
Family has a member with long-lasting cough (>3 months)						
Yes	3 (0.6)	0 (0)	NA			
No	480 (99.4)	26 (5.4)				
House size (m^2^)	*n* = 482			*n* = 490		
Less than 80	278 (60.0)	12 (4.3)	Reference	438 (89.4)	38 (8.7)	Reference
81 or more	204 (40.0)	14 (6.9)	1.73 (0.77–3.88)	52 (10.6)	5 (9.6)	1.08 (0.40–2.89)

PT: pertussis toxin; IgG: immunoglobulin G; * adjusted by sex and age group; ** adjusted by sex; *** adjusted by age group.

**Table 4:** Ratio of anti-PT IgG in 2019 to anti-PT IgG in 2017 and adjusted coefficient of log-transformed ratio for each demographic, clinical, and social characteristic by linear regression.

Characteristics	Number (%)	Ratio of IgG in 2019 to IgG in 2017 Geometric Mean (95% CI)	Adjusted Coefficient *
**All**	*n* = 306	1.45 (1.29–1.62)	Intercept: 0.95 (0.51–1.38)
**Demographics**			
Age group (age in 2017)			
0–2	18 (5.9)	0.86 (0.48–1.52)	−0.31 (−0.85–0.24) **
3–5	45 (14.7)	1.11 (0.87–1.42)	−0.43 (−0.80–−0.06) **
6–15	65 (21.2)	1.72 (1.27–2.33)	−0.09 (−0.43–0.24) **
16–25	52 (17.0)	1.56 (1.11–2.18)	−0.15 (−0.51–0.21) **
26–35	59 (19.3)	1.26 (1.06–1.51)	−0.33 (−0.67–0.01) **
36–55	67 (21.9)	1.78 (1.41–2.25)	Reference
Sex			
Male	128 (41.8)	1.59 (1.31–1.93)	Reference
Female	178 (58.2)	1.35 (1.17–1.56)	−0.20 (−0.42–0.03) ***
**Baseline IgG**			
≥62.5 IU/ml	20 (6.5)	0.45 (0.29–0.68)	−1.21 (−1.69–−0.73) ****
<62.5 IU/ml	286 (93.5)	1.57 (1.40–1.76)	Reference
**Clinical information: Symptoms and medication in preceding two weeks in the 2017 survey**			
Cough			
Yes	46 (15.0)	1.79 (1.14–2.81)	0.44 (0.12–0.76)
No	260 (85.0)	1.39 (1.25–1.56)	Reference
Runny nose			
Yes	45 (14.7)	1.44 (1.00–2.09)	0.14 (−0.19–0.47)
No	261 (85.3)	1.45 (1.28–1.63)	Reference
Difficulty breathing			
Yes	1 (0.3)	3.05 (NA)	0.53 (−1.42–2.47)
No	305 (99.7)	1.44 (1.28–1.62)	Reference
Took antibiotics			
Yes	21 (6.9)	1.43 (0.82–2.51)	0.08 (−0.36–0.53)
No	285 (93.1)	1.45 (1.29–1.63)	Reference
**Clinical information: History and underlying condition**			
Ever diagnosed with Pertussis			
Yes	1 (0.3)	0.79 (NA)	−0.46 (−2.41–1.48)
No	305 (99.7)	1.45 (1.29–1.63)	Reference
Ever had a persistent cough			
Yes	8 (2.6)	2.02 (0.78–5.24)	0.29 (−0.41–0.99)
No	298 (97.4)	1.43 (1.27–1.61)	Reference
DPT history orally reported			
At least one dose	146 (47.7)	1.36 (1.13–1.63)	0.05 (−0.30–0.40)
No history or unknown	160 (52.3)	1.53 (1.32–1.78)	Reference
DPT vaccine history confirmed			
At least one dose	104 (94.6)	1.23 (1.01–1.50)	−0.97 (−1.83–−0.12)
No history	6 (5.5)	3.71 (0.58–23.87)	Reference
Going to nursery or school			
Yes	140 (45.8)	1.35 (1.13–1.62)	−0.26 (−0.68–0.16)
No	166 (54.3)	1.53 (1.32–1.78)	Reference
Chronic disease			
Yes	10 (3.3)	1.52 (0.90–2.58)	−0.04 (−0.67–0.59)
No	296 (96.7)	1.44 (1.28–1.63)	Reference
Smoking			
Yes	19 (6.2)	2.06 (1.29–3.29)	0.27 (−0.24–0.78)
No	287 (93.8)	1.41 (1.25–1.59)	Reference
**Travel history**			
Traveled to another province in Vietnam since 2012			
Yes	41 (13.4)	1.22 (0.98–1.51)	0.23 (−0.10–0.56)
No	265 (86.6)	1.48 (1.30–1.69)	Reference
Traveled to another country since 2012			
Yes	7 (2.3)	1.13 (0.57–2.22)	0.03 (−0.72–0.78)
No	299 (97.7)	1.45 (1.29–1.64)	Reference
**Household**			
Family with >4 members			
≥5	170 (55.6)	1.38 (1.19–1.60)	−0.00 (−0.23–0.22)
1–4	136 (44.4)	1.53 (1.27–1.84)	Reference
Family has child(ren) aged <12 years			
Yes	170 (55.6)	1.39 (1.21–1.61)	0.01 (−0.23–0.26)
No	136 (44.4)	1.52 (1.25–1.83)	Reference
Family has smoker(s)			
Yes	162 (52.9)	1.35 (1.16–1.57)	−0.09 (−0.31–0.14)
No	144 (47.1)	1.56 (1.30–1.87)	Reference
Family has a member with long-lasting cough (>3 months)			
Yes	2 (0.7)	3.04 (0.00–2062.88)	0.47 (−0.91–1.86)
No	304 (99.4)	1.44 (1.28–1.62)	Reference
House size (m^2^) (*n* = 482)			
15–80	173 (56.7)	1.35 (1.17–1.57)	Reference
81–600	132 (43.3)	1.58 (1.31–1.90)	0.13 (−0.09–0.36)

PT: pertussis toxin; IgG: immunoglobulin G; DPT: diphtheria–pertussis–tetanus vaccine; * adjusted by sex, age group, and baseline IgG (≥62.5 IU/mL); ** adjusted by sex and baseline IgG (≥62.5 IU/mL); *** adjusted by age group and baseline IgG (≥62.5 IU/mL); **** adjusted by age group and sex.

## Data Availability

The database of the study can become available upon contacting the corresponding author of this paper.

## Supplementary Materials

The following supporting information can be downloaded at: https://www.mdpi.com/article/10.3390/vaccines12030225/s1, Table S1: Demographic, clinical, and social characteristics of all the participants and those with anti-PT IgG ≥62.5 IU/ml and crude odds ratios for anti-PT IgG ≥62.5 IU/ml in each characteristic by logistic regression; Table S2: Ratio of anti-PT IgG in 2019 to anti-PT IgG in 2017 and crude coefficient of log-transformed ratio in each demographic, clinical, and social characteristic by linear regression; Table S3: Ratio of anti-PT IgG in 2019 to anti-PT IgG in 2017 and crude and adjusted coefficient of the ratio in each demographic, clinical, and social characteristic by linear regression.
