# Supplementary material for: Sero and Carriage Epidemiology of Pertussis in Urban and Rural Regions in Vietnam
Source: Vaccines (Basel). 2024 Feb 23;12(3):225. doi: 10.3390/vaccines12030225 (PMC10974496; doi:10.3390/vaccines12030225)
Supplement: Supplementary file 1 [file vaccines-12-00225-s001.zip › vaccines-2850969-supplementary.pdf]

Supplementary Table S1. Demographic, clinical, and social characteristics of all the participants and those with anti-PT IgG ≥62.5 IU/ml and crude odds ratios for anti-PT IgG ≥62.5 IU/ml in each characteristic by logistic regression

	Nha Trang in 2017			Quang Ngai in 2019		
Characteristics	All Number (%)	IgG ≥62.5IU/ml N (%)	Crude odds ratio	All Number (%)	IgG ≥62.5IU/ml N (%)	Crude odds ratio
	n=483	n=26		n=1129	n=104	
**Demographics**						
Age group						
3-5	73 (15.1)	7 (9.6)	2.65 (0.75-9.42)	182 (16.1)	18 (9.9)	1.03 (0.52-2.04)
6-15	107 (22.2)	6 (5.6)	1.49 (0.41-5.42)	299 (26.5)	34 (11.4)	1.21 (0.67-2.19)
16-25	105 (21.7)	7 (6.7)	1.79 (0.51-6.29)	158 (14.0)	12 (7.6)	0.77 (0.36-1.65)
26-35	94 (19.5)	2 (2.1)	0.54 (0.10-3.04)	292 (25.9)	21 (7.2)	0.73 (0.38-1.40)
36-55	104 (21.5)	4 (3.9)	reference	198 (17.5)	19 (9.6)	reference
Sex						
Male	207 (42.9)	15 (7.3)	reference	558 (49.4)	43 (7.7)	reference
Female	276 (57.1)	11 (4)	0.53 (0.24-1.18)	571 (50.6)	61 (10.7)	1.43 (0.95-2.16)
**Clinical information: Recent symptoms and medication**						
Respiratory symptom	in proceeding two weeks			in proceeding one month, n=1128		
Yes			3.70 (1.61-8.51)	270 (23.9)	28 (10.4)	0.84 (0.53-1.33)
No			reference	858 (76.1)	76 (8.9)	reference
Cough						
Yes	60 (12.4)	9 (15.0)	4.21 (1.79-9.95)			
No	423 (87.6)	17 (4.0)	reference			
Runny nose						
Yes	58 (12)	4 (6.9)	1.36 (0.45-4.09)			
No	425 (88)	22 (5.2)	reference			
Difficulty breathing						
Yes	2 (0.4)	0 (0.0)	NA			
No	481 (99.6)	26 (5.4)				
Took antibiotics				in proceeding one month, n=1118		
Yes	28 (5.8)	5 (17.9)	4.49 (1.55-12.99)	211 (18.9)	22 (10.4)	0.83 (0.51-1.37)
No	455 (94.2)	21 (4.6)	reference	907 (81.1)	80 (8.8)	reference
**Clinical information: History and underlying condition**						
Ever diagnosed with Pertussis	n=291 (asked in 2019)			n=1057		
Yes	1 (0.3)	0 (0.0)	NA	3 (0.3)	2 (66.7)	20.19 (1.81-224.72)
No	290 (99.7)	12 (4.1)		1054 (99.7)	95 (9.0)	reference
Ever had persistent cough	n=291 (asked in 2019)			n=1073		
Yes	8 (2.8)	0 (0.0)	NA	10 (0.9)	3 (30.0)	4.22 (1.07-16.58)
No	283 (97.3)	12 (4.2)		1063 (99.1)	98 (9.2)	reference
DPT history orally reported						
At least one dose	204 (42.2)	12 (5.9)	0.37 (0.10-1.39)			
No history or unknown	279 (57.8)	14 (5.0)	reference			

DPT vaccine history confirmed	n=95 (confirmed in 2019)			n=291		
At least one dose	90 (94.7)	5 (5.3)	NA	275 (94.5)	30 (10.9)	0.86 (0.19-3.96)
No DPT history	5 (5.3)	0 (0.0)		16 (5.5)	2 (12.5)	reference
Going to nursery or school						
Yes	214 (44.3)	18 (8.4)	4.2 (0.82-21.49)	382 (33.8)	42 (11.0)	1.37 (0.90-2.07)
No	269 (55.7)	8 (3)	reference	748 (66.2)	62 (8.3)	reference
Chronic disease						
Yes	18 (3.7)	1 (5.6)	0.81 (0.10-6.51)	159 (13.7)	15 (9.4)	1.03 (0.58-1.83)
No	465 (96.3)	25 (5.4)	reference	970 (86.3)	89 (9.2)	reference
Smoking						
Yes	28 (5.8)	2 (7.1)	1.71 (0.31-9.39)	155 (13.7)	16 (10.3)	1.16 (0.66-2.03)
No	455 (94.2)	24 (5.3)	reference	974 (86.3)	88 (9.0)	reference
**Travel history**						
Traveled to other province in Vietnam since 2012	n=291 (asked in 2019)			n=1128		
Yes	39 (13.4)	2 (5.1)	0.86 (0.18-4.21)	105 (9.3)	6 (5.7)	1.75 (0.75-4.09)
No	252 (86.6)	10 (4.0)	reference	1023 (90.7)	98 (9.6)	reference
Traveled to other country since 2012	n=291 (asked in 2019)			n=1128		
Yes	6 (2.1)	1 (16.7)	0.24 (0.02-2.42)	7 (0.6)	0 (0)	NA
No	285 (97.9)	11 (3.9)	reference	1121 (99.4)	104 (9.3)	
**Household**						
Family with >4 members						
≥5	262 (54.2)	14 (5.3)	0.98 (0.44-2.23)	569 (50.4)	55 (9.7)	1.12 (0.75-1.67)
1-4	221 (45.8)	12 (5.4)	reference	561 (49.7)	49 (8.7)	reference
Family has child(ren) aged <12 years						
Yes	258 (53.4)	13 (5.0)	1.01 (0.44-2.34)	995 (88.1)	94 (9.5)	1.30 (0.66-2.57)
No	225 (46.6)	13 (5.8)	reference	135 (12)	10 (7.4)	reference
Family has smoker(s)				n=1127		
Yes	253 (52.4)	15 (5.9)	1.22 (0.54-2.76)	659 (58.5)	63 (9.6)	0.91 (0.60-1.37)
No	230 (47.6)	11 (4.8)	reference	468 (41.5)	41 (8.8)	reference
Family has a member with long lasting cough (>3 months)						
Yes	3 (0.6)	0 (0.0)	NA			
No	480 (99.4)	26 (5.4)				
House size (meter^2)	n=482			n=490		
Less than 80	278 (60.0)	12 (4.3)	reference	438 (89.4)	38 (8.7)	reference
81 or more	204 (40.0)	14 (6.9)	1.73 (0.77-3.88)	52 (10.6)	5 (9.6)	1.12 (0.42-2.98)

PT; pertussis toxin, IgG; immunoglobulin G

Supplementary Table S2. Ratio of anti-PT IgG in 2019 to anti-PT IgG in 2017 and crude coefficient of log-transformed ratio in each demographic, clinical, and social characteristic by linear regression

Characteristics	Number (%)	Ratio of IgG in 2019 to IgG in 2017<br>Geometric mean (95% CI)	Coefficient
**All**	n=306	1.45 (1.29-1.62)	Intercept: 0.37 (0.25-0.48)
**Demographics**			
Age group (age in 2017)			
0-2	18 (5.9)	0.86 (0.48-1.52)	−0.73 (−1.26-−0.20)
3-5	45 (14.7)	1.11 (0.87-1.42)	−0.47 (−0.86-−0.09)
6-15	65 (21.2)	1.72 (1.27-2.33)	−0.04 (−0.38-0.31)
16-25	52 (17.0)	1.56 (1.11-2.18)	−0.14 (−0.50-0.23)
26-35	59 (19.3)	1.26 (1.06-1.51)	−0.34 (−0.70-0.01)
36-55	67 (21.9)	1.78 (1.41-2.25)	reference
Sex			
Male	128 (41.8)	1.59 (1.31-1.93)	reference
Female	178 (58.2)	1.35 (1.17-1.56)	−0.16 (−0.40-0.07)
**Baseline IgG**			
≥62.5 IU/ml	20 (6.5)	0.45 (0.29-0.68)	−1.26 (−1.70-−0.81)
<62.5 IU/ml	286 (93.5)	1.57 (1.40-1.76)	reference
**Clinical information: Symptoms and medication in preceding two weeks in the 2017 survey**			
Cough			
Yes	46 (15.0)	1.79 (1.14-2.81)	0.25 (−0.07-0.57)
No	260 (85.0)	1.39 (1.25-1.56)	reference
Runny nose			
Yes	45 (14.7)	1.44 (1.00-2.09)	−0.00 (−0.33-0.32)
No	261 (85.3)	1.45 (1.28-1.63)	reference
Difficulty breathing			
Yes	1 (0.3)	3.05 (NA)	0.75 (−1.28-2.78)
No	305 (99.7)	1.44 (1.28-1.62)	reference
Took antibiotics			
Yes	21 (6.9)	1.43 (0.82-2.51)	−0.01 (−0.47-0.45)
No	285 (93.1)	1.45 (1.29-1.63)	reference
**Clinical information: History and underlying condition**			
Ever diagnosed with Pertussis			
Yes	1 (0.3)	0.79 (NA)	−0.60 (−2.63-1.43)
No	305 (99.7)	1.45 (1.29-1.63)	reference
Ever had persistent cough			
Yes	8 (2.6)	2.02 (0.78-5.24)	0.34 (−0.39-1.07)
No	298 (97.4)	1.43 (1.27-1.61)	reference

DPT history orally reported			
At least one dose	146 (47.7)	1.36 (1.13-1.63)	−0.12 (−0.35-0.11)
No history or unknown	160 (52.3)	1.53 (1.32-1.78)	reference
DPT vaccine history confirmed			
At least one dose	104 (94.6)	1.23 (1.01-1.50)	−1.10 (−1.98-−0.22)
No history	6 (5.5)	3.71 (0.58-23.87)	reference
Going to nursery or school			
Yes	140 (45.8)	1.35 (1.13-1.62)	−0.12 (−0.35-0.11)
No	166 (54.3)	1.53 (1.32-1.78)	reference
Chronic disease			
Yes	10 (3.3)	1.52 (0.90-2.58)	0.05 (−0.60-0.71)
No	296 (96.7)	1.44 (1.28-1.63)	reference
Smoking			
Yes	19 (6.2)	2.06 (1.29-3.29)	0.38 (−0.10-0.86)
No	287 (93.8)	1.41 (1.25-1.59)	reference
**Travel history**			
Traveled to other province in Vietnam since 2012			
Yes	41 (13.4)	1.22 (0.98-1.51)	0.20 (−0.14-0.54)
No	265 (86.6)	1.48 (1.30-1.69)	reference
Traveled to other country since 2012			
Yes	7 (2.3)	1.13 (0.57-2.22)	0.25 (−0.52-1.03)
No	299 (97.7)	1.45 (1.29-1.64)	reference
**Household**			
Family with >4 members			
≥5	170 (55.6)	1.38 (1.19-1.60)	−0.10 (−0.33-0.13)
1-4	136 (44.4)	1.53 (1.27-1.84)	reference
Family has child(ren) aged <12 years			
Yes	170 (55.6)	1.39 (1.21-1.61)	−0.09 (−0.32-0.15)
No	136 (44.4)	1.52 (1.25-1.83)	reference
Family has smoker(s)			
Yes	162 (52.9)	1.35 (1.16-1.57)	−0.14 (−0.38-0.09)
No	144 (47.1)	1.56 (1.30-1.87)	reference
Family has a member with long lasting cough (>3 months)			
Yes	2 (0.7)	3.04 (0.00-2062.88)	0.75 (−0.69-2.19)
No	304 (99.4)	1.44 (1.28-1.62)	reference
House size (meter^2) (n=482)			
Less than 80	173 (56.7)	1.35 (1.17-1.57)	reference
81 or more	132 (43.3)	1.58 (1.31-1.90)	0.15 (−0.08-0.39)

PT; pertussis toxin, IgG; immunoglobulin G, DPT; diphtheria-pertussis-tetanus vaccine

Supplementary Table S3. Ratio of anti-PT IgG in 2019 to anti-PT IgG in 2017 and crude and adjusted coefficient of the ratio in each demographic, clinical, and social characteristic by linear regression

Characteristics	Number (%)	Coefficient	Adjusted coefficient*
**All**	n=306	Intercept: 3.37 (2.22-4.51)	Intercept: 6.14 (1.62-10.65)
**Demographics**			
Age group (age in 2017)			
0-2	18 (5.9)	−1.79 (−7.08-3.50)	−2.23 (−7.56-3.09)**
3-5	45 (14.7)	−1.82 (−5.66-2.02)	−1.91 (−5.75-1.93)**
6-15	65 (21.2)	2.29 (−1.18-5.76)	2.10 (−1.38-5.58)**
16-25	52 (17.0)	0.44 (−3.25-4.12)	0.38 (−3.30-4.06)**
26-35	59 (19.3)	−1.99 (−5.55-1.57)	−1.78 (−5.35-1.79)**
36-55	67 (21.9)	reference	Reference
Sex			
Male	128 (41.8)	reference	Reference
Female	178 (58.2)	−1.83 (−4.14-0.49)	−1.62 (−3.99-0.74)***
**Baseline IgG**			
≥62.5 IU/ml	20 (6.5)	−2.94 (−7.57-1.68)	−2.40 (−7.42-2.61)
<62.5 IU/ml	286 (93.5)	reference	reference
**Clinical information: Symptoms and medication in preceding two weeks in the 2017 survey**			
Cough			
Yes	46 (15.0)	5.49 (2.35-8.64)	6.25 (3.01-9.49)
No	260 (85.0)	reference	reference
Runny nose			
Yes	45 (14.7)	3.16 (−0.05-6.38)	3.89 (0.50-7.28)
No	261 (85.3)	reference	reference
Difficulty breathing			
Yes	1 (0.3)	−0.32 (−20.39-19.76)	−1.91 (−22.09-18.26)
No	305 (99.7)	reference	reference
Took antibiotics			
Yes	21 (6.9)	0.86 (−3.68-5.39)	0.85 (−3.78-5.48)
No	285 (93.1)	reference	reference
**Clinical information: History and underlying condition**			
Ever diagnosed with Pertussis			
Yes	1 (0.3)	−2.58 (−22.66-17.49)	−0.38 (−20.5-19.74)
No	305 (99.7)	reference	reference
Ever had persistent cough			
Yes	8 (2.6)	1.48 (−5.70-8.66)	1.86 (−5.41-9.12)
No	298 (97.4)	reference	reference

DPT history orally reported			
At least one dose	146 (47.7)	1.18 (−1.11-3.47)	1.93 (−1.69-5.54)
No history or unknown	160 (52.3)	reference	reference
DPT vaccine history confirmed			
At least one dose	104 (94.6)	−20.76 (−31.5-−10.02)	−24.34 (−35.44-−13.23)
No history	6 (5.5)	reference	reference
Going to nursery or school			
Yes	140 (45.8)	0.93 (−1.37-3.23)	−1.05 (−5.38-3.29)
No	166 (54.3)	reference	reference
Chronic disease			
Yes	10 (3.3)	−1.33 (−7.77-5.12)	−2.05 (−8.53-4.42)
No	296 (96.7)	reference	reference
Smoking			
Yes	19 (6.2)	0.53 (−4.22-5.28)	−0.33 (−5.61-4.95)
No	287 (93.8)	reference	reference
**Travel history**			
Traveled to other province in Vietnam since 2012			
Yes	41 (13.4)	2.14 (−1.22-5.49)	2.02 (−1.37-5.42)
No	265 (86.6)	reference	reference
Traveled to other country since 2012			
Yes	7 (2.3)	2.05 (−5.61-9.71)	1.70 (−6.02-9.43)
No	299 (97.7)	reference	reference
**Household**			
Family with >4 members			
≥5	170 (55.6)	−0.53 (−2.84-1.77)	−0.21 (−2.56-2.15)
1-4	136 (44.4)	reference	reference
Family has child(ren) aged <12 years			
Yes	170 (55.6)	−0.47 (−2.77-1.84)	0.43 (−2.09-2.95)
No	136 (44.4)	reference	reference
Family has smoker(s)			
Yes	162 (52.9)	−1.56 (−3.85-0.73)	−1.50 (−3.82-0.81)
No	144 (47.1)	reference	reference
Family has a member with long lasting cough (>3 months)			
Yes	2 (0.7)	0.08 (−14.14-14.30)	−3.31 (−17.68-11.05)
No	304 (99.4)	reference	reference
House size (meter^2) (n=482)			
Less than 80	173 (56.7)	reference	reference
81 or more	132 (43.3)	0.66 (−1.66-2.98)	0.50 (−1.84-2.84)

PT; pertussis toxin, IgG; immunoglobulin G, DPT; diphtheria-pertussis-tetanus vaccine

* adjusted by sex and age group
** adjusted by sex
*** adjusted by age group
